# Expression and significance of miR-30d-5p and SOCS1 in patients with recurrent implantation failure during implantation window

**DOI:** 10.1186/s12958-021-00820-2

**Published:** 2021-09-08

**Authors:** Yuhao Zhao, Dongmei He, Hong Zeng, Jiefeng Luo, Shuang Yang, Jingjing Chen, Raed K. Abdullah, Nenghui Liu

**Affiliations:** 1 Reproductive Medical Center, Department of Obstetrics and Gynecology, Xiangya Hospital, Central South University, Changsha, 410008 Hunan China; 2 Department of Obstetrics, Xiangya Hospital, Central South University, Changsha, 410008 Hunan China

**Keywords:** In vitro fertilization, Embryo transfer, Recurrent implantation failure, Endometrial receptivity, MiR-30d-5p, SOCS1

## Abstract

**Background:**

Poor endometrial receptivity is a major factor that leads to recurrent implantation failure. However, the traditional method cannot accurately evaluate endometrial receptivity. Various studies have indicated that microRNAs (miRNAs) are involved in multiple processes of embryo implantation, but the role of miRNAs in endometrial receptivity in patients with recurrent implantation failure (RIF) remains elusive. In the present study, we investigated the presence of pinopodes and the roles of miR-30d-5p, suppressor of cytokine signalling 1 (SOCS1) and the leukaemia inhibitory factor (LIF) pathway in women with a history of RIF during the implantation window.

**Methods:**

Endometrial tissue samples were collected between January 2018 to June 2019 from two groups of women who underwent in vitro fertilisation and embryo transfer (IVF-ET) or frozen ET. The RIF group included 20 women who underwent ≥ 3 ETs, including a total of ≥ 4 good-quality embryos, without pregnancy, whereas the control group included 10 women who had given birth at least once in the past year. An endometrial biopsy was performed during the implantation window (LH + 7). The development of pinopodes in the endometrial biopsy samples from all groups was evaluated using scanning electron microscopy (SEM). Quantitative reverse transcription-polymerase chain reaction and western blotting were used to investigate the expression levels of miR-30d-5p, SOCS1, and the LIF pathway.

**Results:**

The presence of developed pinopodes decreased in patients with RIF on LH + 7. The expression level of miR-30d-5p decreased in the endometria during the implantation window of patients with RIF, whereas the mRNA and protein levels of SOCS1 were significantly higher in the RIF group than in the control group. Furthermore, a negative correlation was observed between the expression of miR-30d-5p and SOCS1 (*r*^2^ = 0.8362). In addition, a significant decrease in LIF and p-STAT3 expression was observed during the implantation window in patients with RIF.

**Conclusions:**

MiR-30d-5p and SOCS1 may be potential biomarkers for endometrial receptivity. Changes in pinopode development and abnormal expression of miR-30d-5p, SOCS1 and LIF pathway in the endometrium could be the reasons for implantation failure.

## Background

Infertility is a global reproductive health problem that affects 10–15% of the reproductive-age couples [1]. After 40 years of development, in vitro fertilization (IVF) and embryo transfer (ET) have become an effective and common therapeutic approaches for couples with infertility [2]. However, in IVF cycles, many couples with infertility fail to achieve pregnancy repeatedly with no signs of embryo implantation or production of HCG. One possible cause of the unsuccessful implantation rate is reduced endometrial receptivity, although the transferred embryos are of high quality [3].

Recurrent implantation failure (RIF) is defined as failure to achieve a clinical pregnancy after transfer of at least four good-quality embryos (score ≥ 7) in a minimum of three fresh or frozen cycles [4]. In some patients with RIF, endometrial receptivity may be impaired, leading to the window of implantation (WOI) being displaced and asynchrony between the functional embryo and endometrium tissue, which may result in implantation failure [5]. Therefore, the assessment of endometrial receptivity is of utmost importance in the IVF procedures to increase the rate of implantation in patients with RIF. During WOI, the endometrium undergoes well-defined morphological and molecular changes that allow embryos to adhere and invade, including the developing pattern of microvilli of the epithelial cells and pinopode growth, and alterations in various signalling molecules and inflammatory cytokines [6]. Despite advances in the understanding of implantation failure, the pathogenesis of RIF is complex and requires a multidimensional explanation.

Pinopodes are mushroom-like protrusions that appear on the apical surface of endometrial luminal epithelial cells. These protrusions are formed at the beginning of the WOI by cell swelling and the fusing of several adjacent microvilli together, and then reach the maximum size [7]. Pinopodes are the ultrastructural markers of endometrial receptivity, as they are likely to be the preferred sites of embryo–endometrium interactions [8]. Furthermore, the appearance of pinopodes is consistent with the expression of other biomarkers of endometrial receptivity in humans, such as leukaemia inhibitory factor (LIF) and its receptor, integrin αVβ3, mucin 1 [9], and homeo-box gene 10 [10].

MicroRNAs (miRNAs) are endogenous non-coding RNAs that regulate gene expression by post-transcriptional degradation and/or translational repression of target messenger RNAs (mRNAs). Various studies have shown that miRNAs orchestrate a large variety of physiological processes in humans and exert critical effects on factors that affect the embryo implantation process [11]. In humans, efforts have been made to describe the miRNA profiles in isolated endometrial epithelial cells during the late proliferative phase and mid-secretory phase, which suggest a new level of suppression of gene expression during epithelial cell proliferation in the receptive endometrium [12]. In recent studies, miRNA profiles were described in patients undergoing IVF in natural and stimulated cycles, and the expression levels of miRNAs were compared between receptive and pre-receptive endometrium [13]. Interestingly, miR-30d was consistently upregulated during the acquisition of endometrial receptivity compared to the pre-receptive stage. However, no studies have investigated the relationship between miR-30d and classic biomarkers of endometrial receptivity, additionally, the expression of miR-30d in the endometria of patients with RIF remains unclear. In the present study, we evaluated the expression patterns of pinopodes on the endometrial surface, and then we investigated the expression level of miR-30d-5p. SOCS1 and LIF pathway in women with RIF during WOI.

## Methods

### Patients and tissues

All study subjects in this study were registered at the Reproduction Medical Center, Xiangya Hospital between January 2018 and June 2019. The study was approved by the Institutional Ethics Committee of Xiangya Hospital of Central South University. All patients signed an informed consent forms before inclusion.

For the control group, endometrial biopsies were obtained from fertile women who underwent the first cycle of IVF treatment for either male or tubal factor infertility (*n* = 10). All of these women had regular menstrual cycles ranging in length from 25 to 35 days, had not used any hormonal treatment for ≥ 3 months before the biopsy, had given birth at least once.

For the RIF group, endometrial biopsies were obtained from women who failed to get pregnant after ≥ 3 ETs, including a total of ≥ 4 good-quality embryos (*n* = 20). The RIF for these women could not be explained in any other way, after a detailed infertility analysis.

The inclusion criteria for both groups were as follows: (1) regular menstrual cycle with cycle length between 25 and 35 days; (2) 25–40 years of maternal age; (3) normal endocrine profile including normal serum level of follicle-stimulating hormone (FSH < 10 mIU/mL), luteinizing hormone (LH < 10 mIU/mL), and estradiol (E2 < 50 pg/mL) on day 3 of the menstrual cycle; (4) normal body mass index (BMI) ranging between 18.5–24.9 kg/m^2^; (5) normal ovulation by ultrasound monitoring and (6) infertility due to either tubal or male factors.

The exclusion criteria for all participants were as follows: (1) endometrial or uterine pathology (adenomyosis, fibroids, endometrial polyps and hyperplasia, endometriosis, and chronic endometritis), (2) polycystic ovary syndrome, (3) hydrosalpinx, (4) couples with abnormal karyotypes, (5) positive lupus anticoagulant or anticardiolipin antibodies, (6) endocrine disease (abnormal blood glucose level or thyroid dysfunction, hyperprolactinemia, diminished ovarian reserve, and premature ovarian failure), and (7) use of any contraceptive drugs or intrauterine devices within the last 6 months. The exclusion criteria were monitored using various approaches such as vaginal ultrasonography, hysteroscopy, laparoscopy, karyotyping analysis, and relevant hormone and immunological tests.

### Collection of endometrial biopsies

Follicle development was monitored using transvaginal ultrasound from day 10 of menstruation onwards identification of a dominant follicle ≥ 15 mm, and serum LH and E2 levels were quantified daily. The day on which LH level peaked (≥ 20 mIU/mL) was considered as the day of LH + 0, and the WOI was defined as the day of LH + 7. Endometrial specimens were collected in a cycle before ET cycle using a Pipelle sampler (Prodimed, France) during the implantation period on the 7th day after the LH surge (LH + 7). The obtained endometrial tissues were immediately sent to the laboratory for further processing within 30 min after the endometrial biopsy. One part was examined the presence of pinopodes by scanning electron microscopy, and the other part was stored at -80 °C until subsequent RNA and protein extraction.

### Scanning electron microscopy (SEM)

To examine the presence of pinopodes in luminal epithelium, one part of the specimens was fixed in 0.1 M PBS (pH 7.4) containing 2.5% glutaraldehyde overnight, The samples were washed twice in buffer containing sodium cacodylate and calcium chloride (pH 7.4) and once in distilled water. Thereafter, they were dehydrated, first in increasing concentrations of ethanol (70, 95, and 99.5%), and then in acetone, and dried in a critical point drier with carbon dioxide. The specimens were mounted on a specimen holder, coated with platinum, and examined using a JEOL 820 scanning electron microscope (JEOL, Tokyo, Japan). Scanning electron micrographs of pinopodes were obtained from 10 randomly selected areas of each tissue specimen and were graded semiquantitatively. Based on the literature, pinopode coverage of the uterine endometrium can be scored as follows: 0 (absence of pinopodes), 1 (covering < 25% of the surface epithelium, few), 2 (covering 25–50% of the surface epithelium, moderate), or 3 (covering > 50% of the surface epithelium, abundant) [14]. A single score was assigned to the most representative photomicrograph of the 10 images obtained for each tissue specimen.

### RNA extraction, reverse transcription, and quantitative polymerase chain reaction (qPCR)

Total RNA was extracted using a standard TRIzol-based protocol (Invitrogen, USA) according to the manufacturer’s instructions. cDNA was synthesised using the RevertAid First Strand cDNA Synthesis Kit (Thermo Fisher Scientific, USA) and miRNA reverse cDNA kit (GeneCopoeia, Guangzhou, China), respectively. Quantitative reverse transcription-PCR (qRT-PCR) was then performed with the Hieff UNICON® qPCR SYBR Green Master Mix (Yeasen) and miScript SYBR Green PCR kit (GeneCopoeia), according to the manufacturers’ protocols. GAPDH was used as an internal control for SOCS1 quantification, and U6 snRNA was used as internal control for miR-30d-5p quantification. All reactions were performed using the ViiA™ 7 Real-Time PCR System (Life Technologies, USA). The primer sequences are shown in Table 1. The 2^−ΔΔCT^ method was used to analyse relative RNA expression levels.

**Table 1:** Primers used in the real time quantitative polymerase chain reaction analysis

	Sequence(5′–3′)
miR-30d-5p	CGGGTGTAAACATCCCCGACTGGAAG
U6	CGCTTCACGAATTTGCGTGTCAT
SOCS1	F: TTCGCCCTTAGCGTGAAGATGG
	R: TAGTGCTCCAGCAGCTCGAAGA
LIF	F: AGATCAGGAGCCAACTGGCACA
	R: GCCACATAGCTTGTCCAGGTTG
GAPDH	F: GTCTCCTCTGACTTCAACAGCG
	R: ACCACCCTGTTGCTGTAGCCAA

### miRNA-mRNA interaction analysis

The interaction between miRNAs and mRNAs was predicted from the crosslinking immunoprecipitation RNA sequencing data obtained using the starBase platform (http://starbase.sysu.edu.cn/).

### Protein preparation and western blotting

Total protein was extracted from snap-frozen endometrial samples. Electrophoresis was performed on a 10% SDS–polyacrylamide gel, and the proteins were transferred onto PVDF membranes. Membranes were blocked in 5% w/v non-fat milk for 1 h, and then incubated at 4 °C overnight with the specific primary antibody for SOCS1 (3950, Cell Signaling Technology, USA, 1:1000), LIF (26,757–1-AP, Proteintech, USA, 1:200), STAT3 (9139, Cell Signaling Technology, USA, 1:1000), and p-STAT3 (9145, Cell Signaling Technology, USA, 1:2000); GAPDH (5174, Cell Signaling Technology, 1:1000) was used as an internal control. After washing the membranes three times with 0.1% Tris-buffered saline with Tween 20, the membranes were incubated with the corresponding secondary antibodies. After multiple washes, target proteins were detected using enhanced chemiluminescence according to the manufacturer’s instructions (Servicebio, Wuhan, China). The integrated light density and grey values were normalised to the values obtained for GAPDH in ImageJ (National Institutes of Health, Bethesda, MD, USA).

### Statistical analysis

Continuous variables are presented as mean ± SD. The differences in continuous variables between the intervention and control groups were analysed using the independent samples *t*-test. The chi-square test was used for the enumeration data. Correlation analysis was performed using Spearman’s correlation test. GraphPad Prism 7 (GraphPad Software) was used for data analysis. Statistical significance was set at *P* < 0.05.

## Results

### Clinical characteristics

Thirty patients were enrolled in this study. Of these, 20 patients with RIF were included in the intervention group, whereas 10 women who had given birth at least once in the previous year were included in the control group. The baseline clinical characteristics of the women in the RIF and control groups are shown in Table 2. There were no significant differences between the two groups in terms of age, years of infertility, BMI, menstrual duration, length of menstrual cycle, and basic serum FSH, LH, estradiol, and anti-Müllerian hormone (AMH) levels. The histological results for each sample showed a normal endometrium at the mid-secretory phase.

**Table 2:** Demographic and reproductive characteristics of participants between two groups

Variables	Control (*n* = 10)	RIF (*n* = 20)	*P* value
Female age (years)	30.80 ± 3.48	33.00 ± 3.62	0.118
Years of infertility (years)	4.65 ± 3.13	3.20 ± 2.82	0.228
BMI (kg/m2)	21.78 ± 2.40	21.54 ± 2.06	0.769
menstrual duration (d)	5.15 ± 1.14	4.70 ± 1.16	0.319
Cycle length (d)	28.80 ± 1.51	28.90 ± 2.13	0.883
FSH (mIU/ml)	6.91 ± 1.41	6.60 ± 1.18	0.549
LH (mIU/ml)	5.62 ± 2.10	4.40 ± 1.18	0.094
E2 (pg/ml)	34.49 ± 10.86	39.02 ± 5.00	0.223
Serum AMH (ng/mL)	4.16 ± 2.69	2.69 ± 1.68	0.127
No. of pregnancy			< 0.001
0	0 (0%)	16 (80%)	
1	3 (30%)	3 (15%)	
≥ 2	7 (70%)	1 (5%)	
No. of live birth			< 0.001
0	0 (0%)	17 (85%)	
1	6 (60%)	3 (15%)	
≥ 2	4 (40%)	0 (0%)	
No. of miscarriage			0.002
0	3 (30%)	18 (90%)	
1	7 (70%)	2 (10%)	
≥ 2	0 (0%)	0 (0%)	

Data are presented as mean ± SD or n (%)

*Control* Receptive control, *RIF* Recurrent implantation failure

### Presence of pinopodes on the endometrial surface

**Fig. 1:**
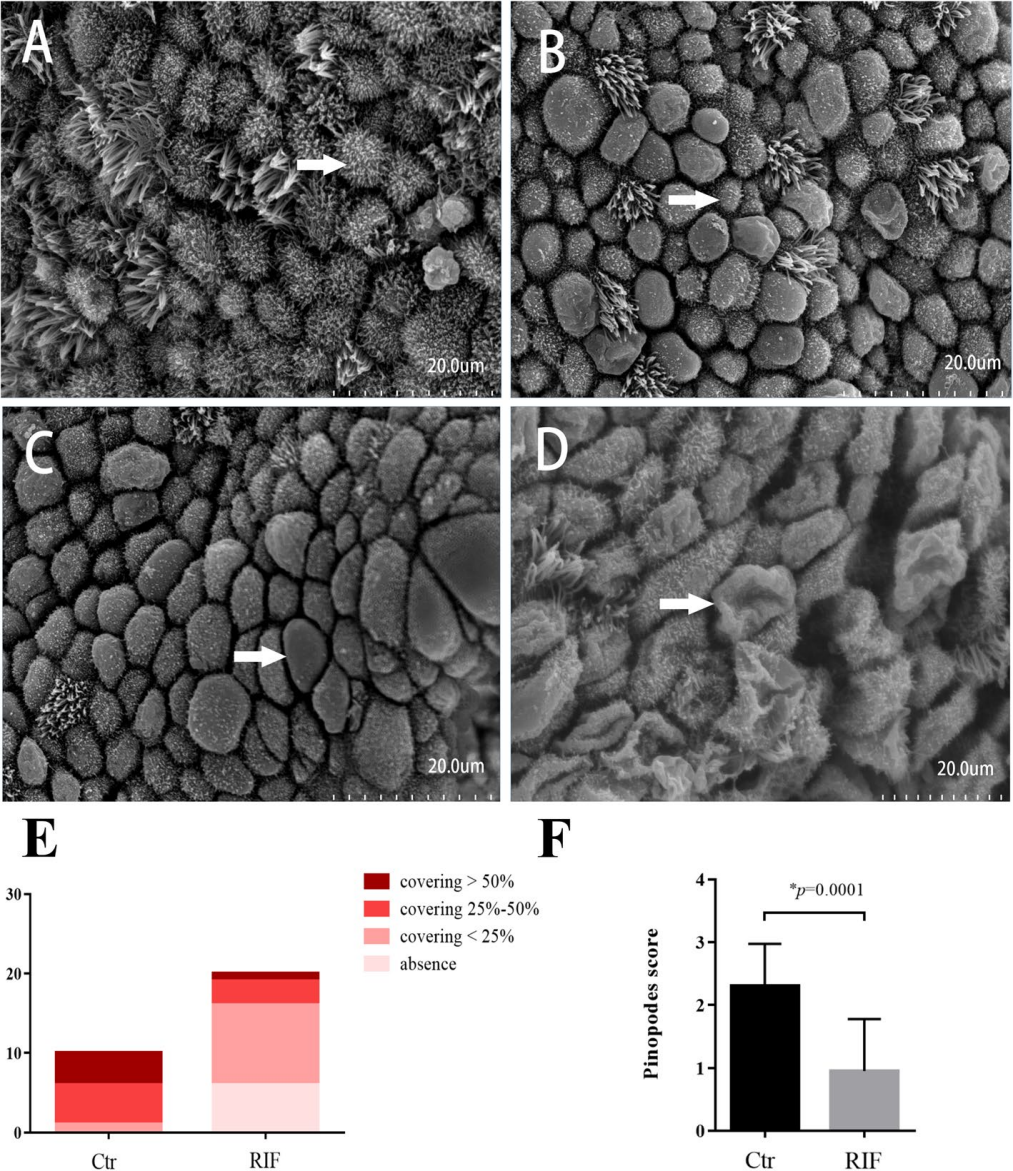
Scanning electron micrographs (SEM) of uterine epithelial tissues from patients on LH + 7. **A** Absence of pinopodes. Secretory cells are covered by microvilli (white arrow). **B** Developing pinopodes. the small semi-spherical projection in the apical membrane is covered by short and sparse microvilli (white arrow). **C** Fully developed pinopodes. Microvilli have disappeared from the projections which appear to be fully maximally distended (white arrow). **D** Regressing pinopodes. The projections have a wrinkled surface (white arrow); small microvilli tips reappeared on the membranes. **E** Comparison of the proportion of the various coverage of pinopodes between the two groups. **F** SEM of pinopodes were graded semi-quantitatively. Control: receptive control (*n* = 10); RIF: recurrent implantation failure (*n* = 20)

Figure 1A-D depict the different presence of pinopodes in the luminal epithelium under SEM on day LH + 7 in the control and RIF groups. Figure 1E show the proportions of developed pinopodes coverage in the control and RIF groups. In the control group, there were 0 case with absence of pinopodes (0%), 1 case with few pinopodes (10%), 5 cases with moderate pinopodes (50%) and 4 cases with abundant pinopodes (40%). In the RIF group, we observed 6 cases with absence of pinopodes (30%), 10 cases with few pinopodes (50%), 3 cases with moderate pinopodes (15%) and 1 case with abundant pinopodes (5%). As shown in Fig. 1F, the pinopodes score of the control group was significantly higher than that of RIF group (*p* = 0.002). This finding indicated that RIF patients had fewer developed pinopodes in the endometria on LH + 7.

### Prediction of the interaction between target mRNA and miR-30d-5p

**Fig. 2:**
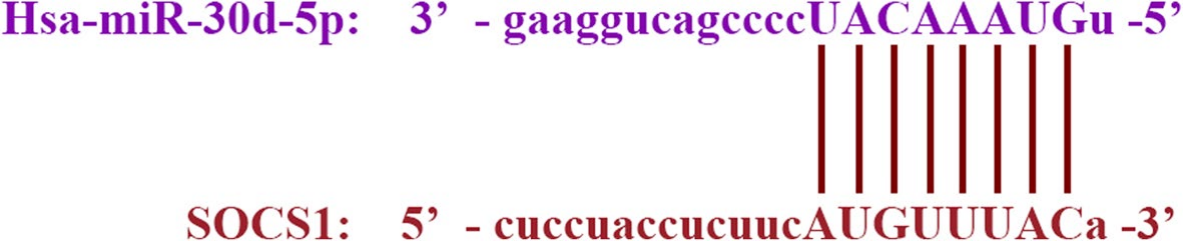
Schematic of the 3′-UTR of SOCS1 with the predicted binding site for has-miR-30d-5p

It is known that miRNAs can bind to their target sequences in the 3ʹ-UTR of mRNAs. To verify whether miR-30d-5p can enhance the degradation of a certain mRNA, the bioinformatics software starBase 3.0 was used to search for miR-30d-5p targets. SOCS1 was selected as the predicted target with a high score. miR-30d-5p contains the binding site of SOCS1 fragment (Fig. 2).

### Expression of miR-30d-5p and SOCS1 in fertile women and patients with RIF

**Fig. 3:**
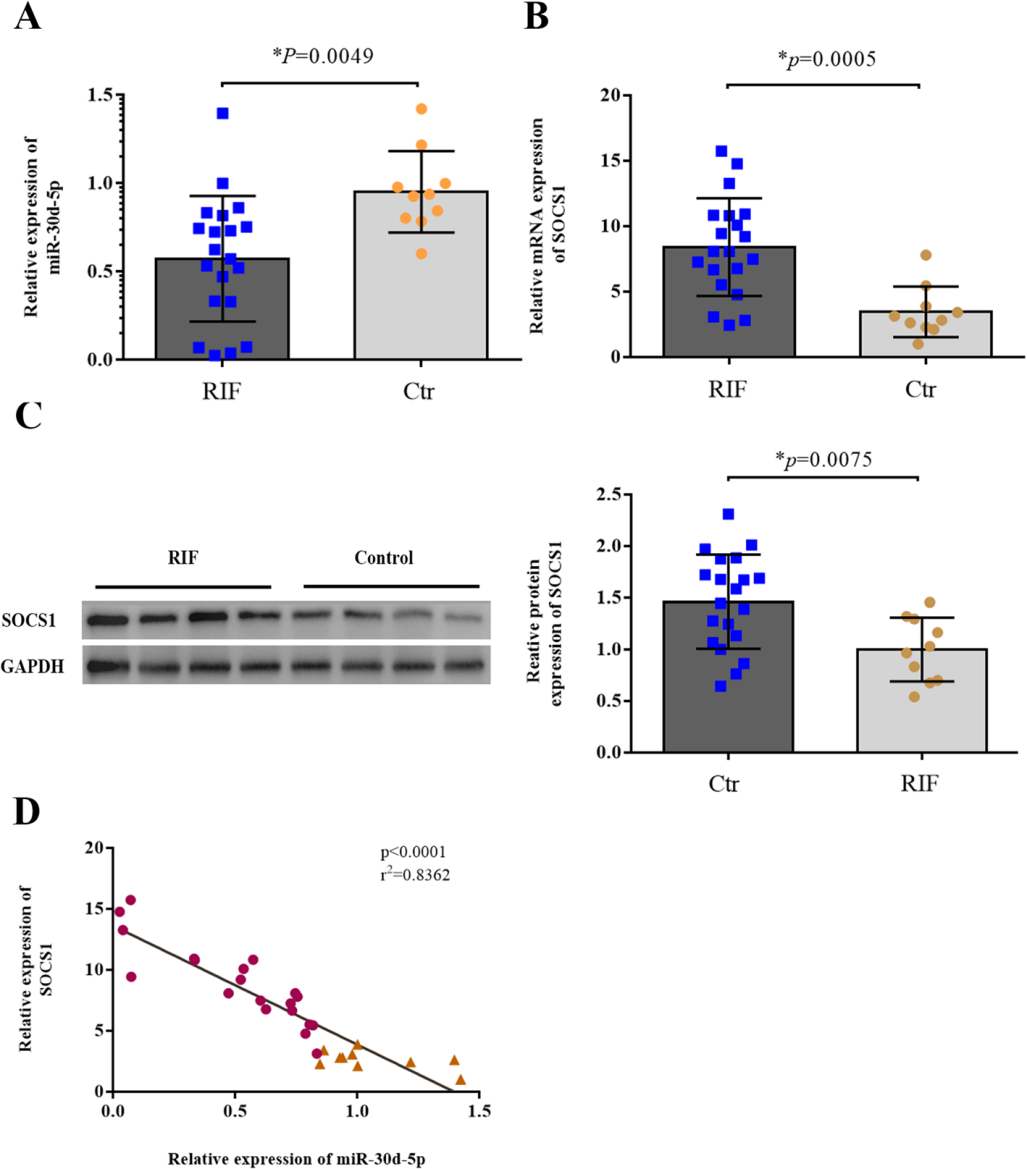
Comparison of miR-30d-5p and SOCS1 expression in endometrium samples from the RIF and control groups. **A**, **B** The relative expression levels of miR-30d-5p and SOCS1 in the RIF group (*n* = 20) and the control group (*n* = 10), as detected via RT-qPCR; U6 was used as an internal control for miR-30d-5p; GAPDH was used as an internal control for SOCS1. **C** The relative expression level of SOCS1 protein in the RIF group (*n* = 20) and the control group (*n* = 10), as detected via western blotting; GAPDH was used as internal control. **D** Spearman correlation suggests a significant negative correlation between the expressions of miR-30d-5p and SOCS1. The Spearman correlation coefficient, *P* values, and sample numbers are indicated on the upper right of the plot. *P < 0.05

Decreased expression of miR-30d-5p was detected in the RIF group, when compared with the control group (*p* = 0.0049, Fig. 3A). In contrast, the mRNA and protein expression of SOCS1 was higher in the RIF group than in the control group (*p* = 0.0005, *p* = 0.0075, Fig. 3B, C). Spearman correlation analysis showed a negative correlation between the expression of miR-30d-5p and SOCS1 at the RNA level (*r*^*2*^ = 0.8362, *p* < 0.0001) (Fig. 3D).

### Expression of LIF, STAT3 and p-STAT3 in fertile women and patients with RIF

**Fig. 4:**
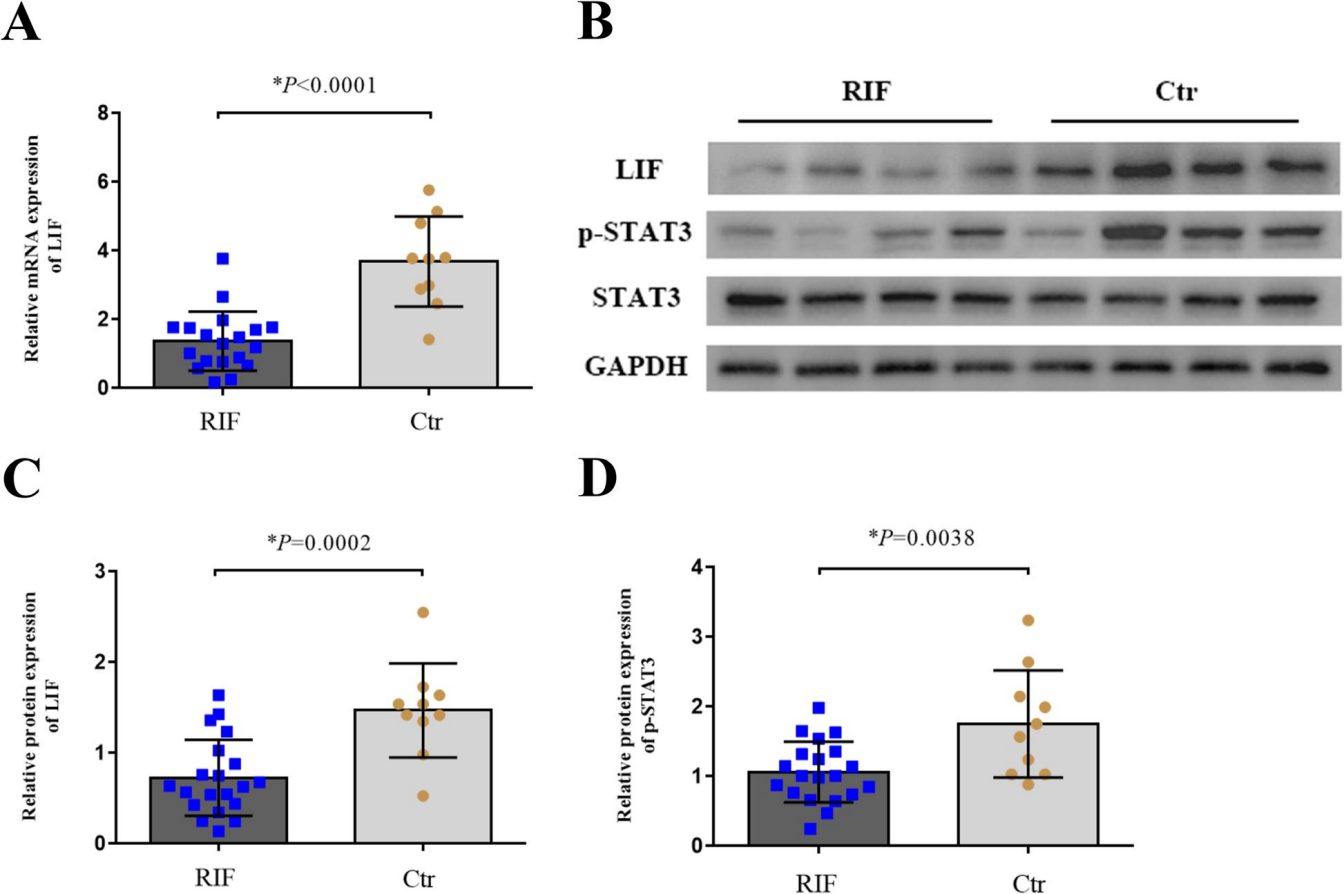
Comparison of miR-30d-5p and SOCS1 expression in endometrium samples from the RIF and control groups. **A** The relative expression level of LIF mRNA in the RIF group (*n* = 20) and the control group (*n* = 10), as detected via RT-qPCR; GAPDH was used as an internal control for LIF. **B** The relative expression levels of LIF, p-STAT3 and STAT3 protein in the RIF group (*n* = 20) and the control group (*n* = 10), as detected via western blotting; GAPDH was used as internal control. **C**, **D** Data of expression of LIF and p-STAT3 protein were expressed as mean ± SD. *P < 0.05

The mRNA levels of LIF were detected in endometrium by RT-qPCR. Data showed that the mRNA levels of LIF were lower in the RIF group than that in the control group (*p* < 0.0001; Fig. 4A). The protein levels of LIF and p-STAT3 were lower in the RIF group than that in the control group (*p* = 0.0002, *p* = 0.0038; Fig. 4B, C and D), and the expression of STAT3 proteins showed no difference between the two groups.

## Discussion

Endometrial receptivity is a hallmark of successful embryo implantation, and poor endometrial receptivity is correlated with infertility [15]. During the menstrual cycle, the endometrium undergoes morphological and molecular changes, allowing for the successful implantation of the embryo. These changes include the appearance of mature pinopodes and the secretion of adhesion molecules and cytokines [15].

In this study, we found that pinopodes were more abundant in the endometria of fertile women than in those of women with RIF, and the pinopode score in fertile women was significantly higher than that in women with RIF. These results are consistent with those of previous studies that suggesting pinopode scoring is a reliable marker of endometrial receptivity and WOI [16, 17]. Pinopode expression is limited to a short period of maximum 2 days in the menstrual cycle corresponding to the window of implantation [18]. It seems that pinopodes are the preferred sites for embryo adhesion and invasion, and the presence of fully developed pinopodes is strongly synchronised with several factors associated with endometrial receptivity, as their temporal and spatial expression [19, 20].

In recent years, various studies have emphasised the significance of miRNAs in endometrial receptivity. For example, the results of animal experiments have shown that miR‐223‐3p suppresses the expression of LIF and pinopodes in the endometrium and may lead to implantation failure [21]. In the endometria of women with PCOS, metformin likely improves endometrial receptivity by downregulating the expression of miR-491-3p and miR-1910-3p, thereby increasing the expression of HOXA10 and ITGB3 [22]. More importantly, many studies have focused on miR-30d, which seems to be a favourable marker of endometrial receptivity. In the study of Satu Kuokkanen et al., it was found that the expression of miR-30d in human late proliferative endometrium was lower than that in the mid-secretory stage [12]. Another study has revealed that polychlorinated biphenyls can impair endometrial receptivity in vitro via regulating miR-30d expression and epithelial-mesenchymal transition [23]. In our study, we demonstrated that miR-30d-5p expression was significantly decreased in the endometria of RIF patients compared to that in fertile women. Then, starbase 3.0 was used to predict and screen out that miR-30d-5p may target SOCS1. On this basis, we detected the expression of SOCS1 in the endometria of women in the RIF and control groups. In contrast, the expression of SOCS1 in the endometria of the RIF group was higher than that in the control group, and a negative correlation was observed between miR-30d-5p and SOCS1. This suggests that high levels of SOCS1 could be a negative factor for embryo implantation.

Exogenous expression of SOCS1 is the key negative regulator of cytokine and growth factor signaling [24]. The SOCS1 protein is rapidly transcribed in response to intracellular Janus kinase-signal transducer and activator of transcription (JAK-STAT) signaling, a cascade governing biological functions including cytokine-induced immunological responses and reproductive processes [25, 26]. In the female endometrium, SOCS1 completely abolishes the activation of the LIF-induced pathway. It has been revealed that compared to fertile women, women with unexplained infertility have high endometrial levels of the inhibitor of LIF, SOCS1, accompanied by low levels of LIF receptor (LIFR) and gp130 [27].

LIF is a pleotropic cytokine which highly correlated with endometrial receptivity and presence of pinopodes. In the human endometrium, LIF expression is relatively low in the proliferative phase, rises after ovulation, and remains high during the mid-luteal phase [28]. LIF is mainly expressed in the glandular and luminal epithelium [29]. It has been found that LIF peak coincide with the appearance of pinopodes, and pinopodes release secretory vesicles that contain LIF in the uterine cavity to enable trophoblast invasion during the WOI [30]. Therefore, we explored the LIF-STAT3 pathway to investigate decreased endometrial receptivity in women with RIF. The expression of both LIF and p-STAT3 protein was found to be significantly lower in the endometrium of women with RIF than in the controls. In a previous study, the isolated epithelium responded to LIF by phosphorylation and nuclear translocation of signal transducer and activator of transcription (STAT3) [31]. Mice that are homozygous for a deletion of the STAT3 or gp130 binding site fail to undergo uterine implantation [32]. Thus, SOCS1 and its function in the LIF pathway play a key role in embryo implantation. It is likely that the function of SOCS1 and LIF are affected in these women with infertility, and these disturbances in the SOCS1 pathway could possibly explain the infertility of women with RIF.

In our study, we detected the expression of miR-30d-5p and SOCS1 on LH + 7. However, in addition to poor endometrial receptivity, the WOI of patients with RIF may have shifted. Therefore, it is necessary to collect the endometrial tissue of RIF patients at multiple time points, such as LH + 5, LH + 7 and LH + 9, and a "gold standard" is required to identify the WOI. Successful implantation of patients or other classic endometrial receptivity markers (LIF, ITGB3, and HOXA10) could serve as standards for identifying WOI. If the embryo is successfully implanted at LH + 5 or LH + 9 with an increase in HCG, it implies that the endometrial WOI has indeed shifted. The molecules that are highly correlated with patient IVF-ET outcomes and classic endometrial receptivity markers may be of more clinical significance. However, if endometrial biopsy is performed at multiple time points in one menstrual cycle, the first uterine cavity operation may affect the microenvironment of the uterine cavity, thereby affecting the gene expression in subsequent biopsy tissues. If the endometrial tissue is collected at multiple time points in different menstrual cycles, there may be differences between different menstrual cycles. Therefore, identifying the relationship between a shifted WOI and endometrial receptivity markers requires further study. In addition, stricter experimental designs and ethical reviews are also required.

Nevertheless, this study has some limitations. First, our data did not show the mechanism how miR-30d-5p downregulates SOCS1. We only conducted tissue experiments to support the hypothesis of the function of miR-30d-5p and SOCS1. Therefore, the hypothesis may still need to be corroborated by further studies wherein cell experiments in vitro would support this hypothesis. Second, although statistically valid, the weakness of our study is the small number of women recruited. However, many previous studies of this nature have involved similar numbers of endometrial samples. Considering the small sample size, we calculated the proper sample size and applied stringent data analysis. Third, it is impossible to infer whether the decreased levels of miR-30d-5p and SOCS1 are the cause of RIF or the result of RIF.

## Conclusions

In conclusion, the abnormal expression of miR-30d-5p and SOCS1, combined with the decreased presence of developed pinopodes, is associated with poor endometrial receptivity and implantation failure. Lower expression of LIF and p-STAT3 in the endometria of women with RIF is suggestive of an impaired LIF pathway, which may be a possible cause of repeated implantation failure.

## Data Availability

The datasets used in the present study are available from the corresponding author upon reasonable request.

## Abbreviations

SOCS1: Suppressor of cytokine signalling 1
LIF: Leukaemia inhibitory factor
FSH: Follicle-stimulating hormone
LH: Luteinizing hormone
E2: Estradiol
STAT3: Signal transducer and activator of transcription
RIF: Recurrent implantation failure
WOI: Window of implantation
IVF: In vitro fertilisation
ET: Embryo transfer
BMI: Body mass index
mRNA: Messenger RNA
miRNA: MicroRNA
SEM: Scanning electron microscopy
PCR: Polymerase chain reaction
